# Mental distress among university students in the Eastern Cape Province, South Africa

**DOI:** 10.1186/s40359-022-00903-8

**Published:** 2022-08-18

**Authors:** Given Mutinta

**Affiliations:** Media and Communication, Nelson Mandela University, Room: 0411, Summerstrand, South Campus, Port Elizabeth, South Africa

**Keywords:** Prevalence, Mental distress, Factors, Students, Universities, Eastern Cape Province

## Abstract

**Introduction:**

Mental distress is a global health concern. Studies show that the prevalence of mental distress is higher in students’ population than in the general population. Therefore, there is need to understand the extent and factors associated with mental distress among students to help policymakers and practitioners in South Africa.

**Aim of the study:**

The study was designed to investigate into the prevalence and factors associated with mental distress among students at universities in the Eastern Cape Province, South Africa.

**Methods:**

A cross-sectional study was conducted among students from four universities in the Eastern Cape Province. The data were collected from 844 students using an online self-reporting questionnaire-20 to assess mental distress of students. Multivariable logistic regression modelling determined the association between socio-demographic and psychosocial factors with students’ mental distress.

**Findings:**

The prevalence of mental distress among students was 53.3% (95% CI 47.0%, 58.1%). Female students were more prone to mental distress than male students ([AOR]: 4.67; 95% CI 2.82, 7.72, *P* = 0.001). Field of study ([AOR]: 3.9; 95% CI 1.74, 5.50, *P* = 0.010), year of study ([AOR]: 4.29; 95% CI 0.86, 21.46, *P* = 0.002), academic workload ([AOR]: 4.66; 95% CI 2.81, 7.71, *P* = 0.003), poor sleep quality ([AOR]: 2.24; 95% CI 1.13, 3.67, *P* = 0.010) and using cannabis ([AOR]: 3.10; 95% CI 1.755, 5.51, *P* = 0.020) were other factors significantly correlated with students’ mental distress.

**Conclusions and recommendations:**

The study found the level of mental distress to be higher among students than what is reported in most of the previous studies on the student population in South Africa. Being a female student, a user of cannabis, and field of study, academic workload and having poor sleep quality were predictors of mental distress. Findings point to the need to devise evidence-driven interventions and strategies to prevent and reduce the problem of mental distress among students.

## Introduction

Mental distress is a substantial disorder of emotional processes, thoughts, or cognition that impairs judgment, behaviour, or ability to cope with the ordinary demands of life [1]. According to the World Health Organisation, mental distress is a huge public health issue affecting society as a whole which involves mental problems such as depression, anxiety and somatic symptoms such as back ache, fatigue, sleeping problems, and headache [1, 2]. Studies have constantly revealed that students have higher levels of mental distress than the general population [1–4]. A study among students in Tanzania and Australia reported significantly higher levels of mental distress than the general population [4, 5].

Studies across the globe including in Asian and sub-Saharan Africa countries have reported higher prevalence of mental distress among university students [3, 6–10]. A study in Saudi Arabia reported 71.9% and in Tanzania 70% [11, 12] mental distress levels among students.

There is a dearth of knowledge on mental distress among students in South African Universities. However, the proportions of mental distress among students in other countries, and in other studies are high, which may also be true among students in South Africa. Knowledge from this study is key to promoting mental health and well-being among students [11, 13].

Studies indicate that students with higher levels of mental distress are prone to experience negative consequences such as significant impaired cognitive functioning [14], poor academic performance [15], learning disabilities [16], and substance abuse linked to risk behaviours [8, 17, 18], higher risk of depression [19], and anxiety disorders [8, 20]. The findings above suggest that mental distress amplifies the risk of other mental health issues.

Previous studies indicate that mental distress among students has been associated with female students reporting higher levels compared to males, lack of interest towards the field of study, not having close friends, examinations, and financial woes [1, 5]. In addition, studies show that disagreements with friends, family history of mental illness, lack of time to rest, use of drugs such as cannabis, limited social support, tight schedules, and lack of extracurricular activities on campuses are associated with students’ mental distress [10, 11].

South Africa, as other low-and middle-income countries is facing numerous challenges in meeting mental health needs, being among one of the least spotlighted areas. This is evident in the shortage of trained personnel in mental health, lack of specific treatment, misplacement of human resource for mental health, problematic insurance coverage for mental disorders, and stigma attached to mental health problems [14]. These challenges have potential to worsen mental health issues especially among students. Therefore, the aim of the study was to ascertain the prevalence and factors associated with mental distress among university students in the Eastern Cape Province, South Africa.

Though reports in South Africa indicate that more than one-third of the students are affected by mental distress at least once during their university life, the issue of mental health has been one of the most lacking health programmes in higher education institutions. For example, higher education institutions lack mental health facilities and trained human resources [9–12]. More important, there is gap on the prevalence and factors associated with mental distress among university students in South Africa. To generate intervention policies and strategies and control mental distress among students, first, it is important to comprehend the extent of mental distress and factors associated with mental distress among students, a sure way of helping practitioners and policymakers in South Africa.

## Methods

### Research design

A cross-sectional study was conducted among students in public universities in the Eastern Cape Province. A cross-sectional research design was used because the design allows studies to collect data to make inferences about a population of interest at one point in time [21]. A cross-sectional research design makes snapshots of the populations about which they gather data. Besides, this design was chosen because it allows studies to collect data from many different individuals at a single point in time [22]. Data were collected from four different public universities in Eastern Cape using quantitative research methodology to quantify the prevalence and factors associated with mental distress among students.

### Sampling technique

The study used purposive sampling method to select respondents. First stage, universities in Eastern Cape namely Nelson Mandela University (NMU), University of Fort Hare (UFH), Rhodes University (RU) and Walter Sisulu University (WSU) were selected using purposive sampling technique. Second stage, all students from the four universities were invited to participate in the study. The questionnaire was posted on students’ respective university communication systems. The questionnaire was posted online together with the consent form describing in detail the purpose of the study. Eight hundred and forty-four (844) students successfully completed the questionnaire.

### Data collection instruments

Data were collected using a structured and pre-tested questionnaire. To achieve the aim of the study and high response rate, eight hundred and forty-four students in the first, second, third-and fourth-year level of study were invited to complete the questionnaire through their respective university communication systems. The survey used a questionnaire with several items. First, sociodemographic characteristics questions were asked on students’ age, sex, year of study, marital status, field of study, residence, history of illness, ever using cannabis, ever consumed alcohol, and sleep quality and quantity. Second, the self-reporting questionnaire (SRQ20) was used to measure the prevalence of mental distress. The SRQ20 was developed by the World Health Organization [14] designed to indicate common mental disorders or mental distress. The tool was validated in low and middle-income countries including South Africa [14]. In this study, students who were found to have eight or more symptoms of SRQ20 questions in the last four weeks were considered as having mental distress. This cut-off point was used based on a validation study of the questionnaire which gave the highest sensitivity and specificity [9]. Third, ever and current substance use were asked. The last part of the questionnaire measured sleep quality and patterns of students using the Pittsburgh sleep quality index which is an effective instrument used to measure the quality and patterns of sleep in adults. The Pittsburgh sleep quality index differentiates poor from good sleep by measuring seven areas: subjective sleep quality, sleep latency, sleep duration, habitual sleep efficiency, sleep disturbances, use of sleeping medications, and daytime dysfunction over the last month [10].

A pilot sample (n = 10) was used to improve the wording and clarity of expression of the survey items. Data from the pilot sample was not used in any further analysis. The final version of the questionnaire required an estimated time of 5–15 min to complete. The questionnaire was posted online together with the consent form describing the purpose of the study in detail. Eight hundred and forty-four questionnaires were collected. The total number of students expected to complete the survey was 120,000. Using the confidence level 95%, population size 120,000 and margin of error 5% the ideal sample size is 384 but this study generated 844 questionnaires expressing more than one hundred per cent response rate. All completed questionnaires were checked for completeness by the investigator.

### Ethical considerations

With adequate knowledge of the study, both male and female students were asked to consent by pressing on the button on the right side of the questionnaire which automatically activated the questionnaire as consent. The questionnaire remained inactive if the consent button was not pressed. Students were informed that participation in the study was voluntary and were at liberty to withdraw from the study anytime without any consequences. Confidentiality, privacy, and anonymity were upheld. The contact details for Nelson Mandela University Research Office were provided in case students had questions. The four universities under study provided gatekeepers’ letters and Nelson Mandela University provided ethical clearance. The questionnaire ran online from July 2021 to August 2021.

### Data analysis

Data were analysed using descriptive statistics that included the computing of percentages and frequencies. Bivariate and multivariable logistic regression analyses were performed, and adjusted odds ratios (AORs) calculated with 95% confidence interval to determine the associations between mental distress and independent variables. All variables with a *P* = value of less than or equal to 0.05 in the bivariate analysis were considered for the final multivariable analysis. A *P*-value less than or equal 0.05 was considered as statistically significant.

## Results

### Demographic descriptions of students

Out of 844 students, 510 (64.0%) were ≤ 21 years of age, and 460 (57.0%) were males as shown in Table 1.

**Table 1:** Demographics of students

Items	Frequency	Percentage
*Sex*		
Female	358	43.0
Male	460	57.0
*Age*		
≤ 21 years	510	64.0
> 21 years	298	36.0
*Marital status*		
Divorced	12	1.5
Married	36	4.4
Single	760	94.1
*Residence*		
Rural	438	54.3
Urban	370	45.7
*University*		
Nelson Mandela University	194	36.1
Rhodes University	172	21.2
University of Fort Hare	192	33.7
Walter Sisulu University	152	30.8
*Year of study*		
1^st^ year	196	24.3
2^nd^ year	304	37.6
3^rd^ year	166	20.5
4^th^ year	142	17.6
*Cumulative Grade Point Average*		
> 3.5	142	18.8
3.00–3.49	258	32.0
2.50–2.99	242	29.0
2.00–2.49	154	19.0
< 1.99	2	0.3
*History of mental distress in the family*		
Yes	198	24.4
No	610	75.6

Four hundred and thirty-eight (54.3%) of students were from rural areas, 760 (94.1%) were single, and 198 (24.4%) reported a family history of mental illness.

### Psycho-social descriptions of students

Alcohol was used by 308 (38%), cannabis 224 (27.6%), and tobacco 74 (9.1%) of the students as presented in Table 2.

**Table 2:** Psychosocial factors among students

Items	Frequency	Percentage
*Sleep duration*		
Greater than 7 h	100	12.4
6 to 7 h	84	10.4
5 to 6 h	372	44.9
< 5 h	262	32.3
*Sleep latency*		
Not during the past month	102	12.6
Less than once a week	338	41.9
Once or twice a week	286	35.3
Three or more times a week	82	10.1
*Day time dysfunction*		
Not during the past month	226	29.3
Less than once a week	338	41.9
Once or twice a week	182	22.6
Three or more times a week	52	6.3
*Sleep efficiency*		
> 85%	302	37.5
75–84%	174	21.6
65–74%	184	22.7
< 65%	158	18.2
*Subjective sleep quality*		
Very good	178	22
Fairly good	244	30.1
Fairly bad	288	35.7
Very bad	96	11.9
*Sleep disturbance*		
Not during the past month	238	29.6
Less than once a week	214	26.4
Once or twice a week	200	24.9
Three or more times a week	156	19.2
*Use of sleep medication*		
Not during the past month	718	89
Less than once a week	50	6.1
Once or twice a week	34	4.1
Three or more times a week	6	0.8
*Sleep quality*		
Good sleep quality	170	20
Poor sleep quality	638	80
*Ever use of cannabis*		
Yes	224	27.6
No	594	72.4
*Current use of cannabis*		
Yes	128	57.2
No	96	42.7
*Frequency of cannabis*		
Once in a week	36	28
Two–three times in a week	62	48.5
More than three times a week	30	23.4
*Ever consume alcohol*		
Yes	308	38
No	500	62
*Currently consuming alcohol*		
Yes	172	55.9
No	136	44.1
*Frequency of alcohol consumption*		
Once in a week	92	53.6
Two–three times in a week	64	37.1
More than three times a week	16	9.3
*Ever smoke tobacco products*		
Yes	74	9.1
No	734	90.9
*Current smoker*		
Yes	32	43.1
No	42	56.8

Most of the students (77.3%) reported that they had less than six hours of sleeping. Results show that 41.9% of students reported daytime dysfunction of less than once in a week and sleep latency. Ninety (11.2%) of students were using sleeping medication, and 738 (79%) were categorised as having poor sleeping quality.

### Prevalence of mental distress among students

The prevalence of mental distress among students was reported to be 53.3% [95% [CI] 0.49, 0.59], 58.2% of female students and 41.8% of male students had mental distress.

### Factors associated with mental distress among students

The multivariable logistic regression analysis indicates that the field of study, year of study, academic workload, sleep quality, using cannabis, and being female were strong factors associated with mental distress among students. Female students showed significant higher levels of mental distress ([AOR]: 4.68; 95% CI 2.82, 7.72, *P* = 0.001) than male students as shown in Table 3.

**Table 3:** Factors associated with mental distress among students

Items	Mental distress		ORs with 95% CI		*P*-Value
	Yes	No	Crude	Adjusted	
*Sex*					
Male	180	284	1.00	1.00	0.701
Female	250	98	3.98 (2.7, 6.07)	4.67 (2.82, 7.72)	0.001
*Age*					
≤ 21 years	138	60	1.98 (1.23, 3.17)	1.69 (0.97, 2.96)	0.701
> 21 years	310	300	1.00	1.00	0.801
*Year of study*					
1^st^ year	160	36	7.1 (3.92, 13.13)	4.29 (0.86, 21.46)	0.200
2^nd^ year	142	152	1.62 (1.03, 2.55)	0.79 (0.18, 3.66)	0.970
≥ 3^rd^ year	118	190	1.00	1.00	0.779
*School workload*					
Yes	258	90	3.97 (2.6, 6.06)	4.66 (2.81, 7.71)	0.300
No	184	280	1.00	1.00	0.760
*Field of study*					
Science	142	80	1.74 (1.11, 2.73)	3.9 (1.74, 5.5)	0.100
Non-science	296	290	1.00	1.00	0.990
*Marital status*					
Yes	134	64	1.96 (1.21, 3.15)	1.67 (0.95, 2.94)	0.870
No	305	305	1.00	1.00	0.790
*Residence*					
Yes	135	63	1.95 (1.18, 3.17)	1.64 (0.92, 2.91)	0.570
No	301	309	1.00	1.00	0.890
*University*					
Yes	137	61	1.92 (1.17, 3.11)	1.63 (0.91, 2.90)	0.980
No	317	293	1.00	1.00	0.670
*History of mental illness in the family*					
Yes	130	68	1.99 (1.24, 3.18)	1.70 (0.98, 2.97)	0.577
No	300	310	1.00	1.00	0.694
*Ever used cannabis*					
Yes	140	82	1.75 (1.12, 2.74)	3.10 (1.75, 5.6)	0.020
No	290	296	1.00	1.00	0.793
*Ever consumed alcohol*					
Yes	146	162	0.70 (0.59, 1.04)	0.9 (0.56, 1.48)	0.557
No	284	216	1.00	1.00	0.660
*Sleep quality and quantity*					
Good sleep quality	64	116	1.00	1.00	0.870
Poor sleep quality	376	272	2.24 (1.37, 3.67)	2.03 (1.13, 3.67)	0.010

The prevalence of mental distress was significantly higher (AOR: 2.03; 95% CI 1.13, 3.67, *P* = 0.010) among students who reported poor sleep quality than their counterpart. Students who use Cannabis had higher likelihood (AOR: 3.10; 95% CI 1.75, 5.51, *P* = 0.020) of experiencing mental distress than students who do not use cannabis.

## Discussion

The study found the prevalence of mental distress to be 53.3%. The finding is higher than results reported in studies conducted in Ethiopia among university students at Adama University that reported 40%, Gondar University 50%, and Hawassa University 52% [12, 15, 16] and study conducted in Norway [20] 22.9%, and Australia [23] 19.2%. The difference observed in findings can be attributed to the explanation that universities in Eastern Cape are in busy towns which increases student's stress [18]. The difference in the level of mental distress can also be attributed to the absence of recreational facilities inside campuses. The finding points to the urgent need to devise interventional programmes designed to prevent and reduce students’ mental distress.

The study found that female students had a higher risk of having mental distress than male students. The current finding agrees with studies conducted in France on psychiatric disorders in students in six universities [24] and Turkey on the prevalence and sociodemographic correlations of depression, anxiety, and stress among university students [25] that found higher levels of mental distress among female students than male students [19, 20, 26]. The possible explanation of the higher prevalence of mental distress among female students is that women are prone to stressors due to hormonal changes during menstruation [23, 27]. Some studies attribute the finding to structural determinants of mental health such as social roles and income of women. The finding points to the need to put in place gender-specific interventional programmes addressing gender-specific risk factors in South African Universities.

The study found that students’ academic workload was associated with mental distress. The finding is supported by studies conducted in the students’ population in South Africa. For example, one study found that academic work demands to be one of the underlying factors to students’ mental distress. In agreement, a study conducted at Gondar University in Ethiopia on mental distress among medical students found academic workload to be associated with mental distress which also caused physical problems such as fatigue, headaches, and digestive complications, and anxiety able to upset the stomach [15].

Being in the first-year level of study was associated with mental distress among students. The finding is supported by a study conducted in Ethiopia among university students at Adama University that reported that students in the first-year level of study experienced a variety of stressors than any other group of students [12]. The finding may be attributed to the finding that first year students are in an important transition to university and the associated increasing academic and social demands they encounter make them susceptible to mental distress. In addition, first year students experience mental distress because they lack the ability to navigate their way and cope with the stress of such a transition consistently identified as having implications for psychosocial wellbeing of first year students. Results from a study conducted among Malaysian university students [28] revealed that factors associated with financial difficulties, demands of the university environment and the university administrative processes, and non-academic related issues were reported as underlying factors to first year students’ mental distress. The finding however on the mental distress of first students presents the opportunity to educate staff and students on the issues associated with the transition to university. The finding provides a platform for educating students regarding issues, such as mental distress, that are typically encountered during first year level of study, and on the importance of developing a range of effective social supports.

There was a positive association between the use of cannabis and mental distress among students. The finding is supported by studies that found that substance use was a predictor of mental distress among students [12, 29–31]. The finding may be attributed to the view that some people use alcohol, caffeine, nicotine, marijuana, and certain pain medicines as a self-regulation strategy to lessen distressful experiences [24, 25, 27, 29, 30, 32]. Other studies explain that people who use substances and have mental disorder have overlapping genetic vulnerability to both disorders [31, 33]. It is therefore important that policymakers, practitioners, and stakeholders should work together to put in place multipronged interventions to lessen the simultaneity of substance use and mental distress among students.

The field of science, in which majority of the students are medical students, reported a higher mental distress level than students from non-science fields. The possible explanation to this finding is that science students include students from the faculties of medicine who are reported by studies to experience higher distress than other students [32]. In agreement, a study in South Africa [34] found that students in the science field especially medical students for their first three years of study mainly do pre-clinical courses including physiology, histology, anatomy, and biochemistry which are more theoretical than practical in design therefore require hours to understand new theoretical material. The next three years students are devoted to building a clinical curriculum and consist of practical work which increases their academic workload worsened by lack of adequate academic supervision.

The study revealed that students with poor sleep quality were more prone to experience mental distress than students with good sleep quality. Similar results were reported by studies conducted among students on psychological wellbeing status among medical and dental students in Makkah in Saudi Arabia [35], a study on sleep quality and its association with psychological distress and sleep hygiene among pre-clinical medical students [36], and a study conducted on correlates of depression, anxiety and stress among Malaysian university students [28]. The current finding can be attributed to the perspective that students with sleep disruptions are more likely to report high levels of stress that can easily change to mental distress [28, 35–37]. Other studies associate the current finding to the view that sleeping disturbance can be either a cause or a symptom of mental distress [36]. For this reason, there is need for a rigorous longitudinal study to explore the link between mental distress and sleep quality among students.

### Limitations of the study

The study employed a cross-sectional research design that does not produce results to confirm a conclusive cause-and-effect relationship. Besides, the SRQ-20 used in this study is a self-report screening tool developed by the World Health Organization specifically for the low-to-middle-income country primary healthcare context but used in the higher education and training setting. Nonetheless, findings generated in this study can be employed as a basis to understand mental distress among students. In addition, the study had a limited scope to comprehensively measure students’ mental distressful experiences. Therefore, there is need to conduct a qualitative study to investigate students’ experiences of mental distress. It is also important to bear in mind that this study used data on students’ self-reported information, making the study likely to be prone to recall bias. To reduce the impact of the limitations of the study, a research instrument validated by a subject expert and standardised by a statistician was used in this study.

## Conclusion

The prevalence of mental distress among students was higher than what is reported in previous studies. Field of study, year of study, academic workload, using cannabis, poor sleep quality, and being female were strong factors associated with mental distress among students.

## Recommendations

First, based on the findings in this study, there is need for the issue of mental distress among students to be given the attention it deserves. Second, there is need for mental distress intervention programmes to target female students, students in the field of science, students in the first year level of study, students reporting heavy academic workload, students who use cannabis, and students who have poor sleep quality associated with mental distress. Third, the study recommends that government and non-government organisations devise evidence-driven interventions and strategies to prevent and reduce the problem of mental distress among students. Mental distress interventions programmes should ensure that students have access to professional mental health services to reduce and prevent their mental distress”.

## Data Availability

All materials in the manuscript, including all relevant raw data, can freely be made available to any person wishing to use them for non-commercial purposes, without breaching participant confidentiality. The datasets used and/or analysed during the present study are available from the corresponding author on reasonable request after all potential articles have been written from the data.

## References

[1] Haile YG, Alemu SM, Habtewold TD (2017). Common mental disorder and its association with academic performance among Debre Berhan university students Ethiopia. Int J Ment Heal Syst.

[2] Vahia VN (2013). Diagnostic and statistical manual of mental disorders 5: a quick glance. Indian J Psychiatry.

[3] Svanum S, Zody ZB (2001). Psychopathology and college grades. J Couns Psychol.

[4] Mane Abhay BKM, Niranjan Paul C, Hiremath SG (2011). Differences in perceived stress and its correlates among students in professional courses. J Clin Diagn Res.

[5] Tavolacci MP, Ladner J, Grigioni S, Richard L, Villet H (2013). Dechelotte P (2013) prevalence and association of perceived stress, substance use and behavioural addictions: a cross-sectional study among university students in France, 2009–2011. BMC Public Health.

[6] Sadeh A, Keinan G, Daon K (2004). Effects of stress on sleep: the moderating role of coping style. Health Psychol.

[7] Sweileh WM, Ali IA, Sawalha AF, Abu-Taha AS, Zyoud SH, Al-Jabi SW (2011). Sleep habits and sleep problems among Palestinian students. Child Adolesc Psychiatry Ment Health.

[8] Cohen S, Evans GW, Stokols D, Krantz DS (1986). Behaviour, health and environmental stress.

[9] Harding TW, de Arango MV, Baltazar JV (1980). Mental disorders in primary health care: a study of their frequency and diagnosis in four developing countries. Psychol Med.

[10] Buysse DJ, Reynolds CF, Monk TH, Berman SR, Kupfer DJ (1989). The Pittsburgh sleep quality index: a new instrument for psychiatric practice and research. Psychiatry Res.

[11] Ng M, Fleming T, Robinson M (2014). Global, regional, and national prevalence of overweight and obesity in children and adults during 1980–2013: a systematic analysis for the global burden of disease study 2013. Lancet.

[12] Dessie Y, Ebrahim J, Awoke T (2013). Mental distress among university students in Ethiopia: a cross sectional survey. Pan Afr Med J.

[13] Samara University, 2018. Students. Retrieved from https://www.su.edu.et/node/23.

[14] WHO (1994). WHO: a users guide to self-reporting questionnaire.

[15] Dachew BA, Azale Bisetegn T, Berhe GR (2015). Prevalence of mental distress and associated factors among undergraduate students of university of Gondar, Northwest Ethiopia: a cross-sectional institutional based study. PLoS ONE.

[16] Tesfaye A (2009). Prevalence and correlates of mental distress among regular undergraduate students of hawassa university: a cross sectional survey. East Afr J Public Health.

[17] Tesfaye M, Hanlon C, Tessema F, Prince M, Alem A (2014). Common mental disorder symptoms among patients with malaria attending primary care in Ethiopia: a cross-sectional survey. PLoS ONE.

[18] Lõhmus M (2018). Possible biological mechanisms linking mental health and heat–a contemplative review. Int J Environ Res Public Health.

[19] Verger P, Combes JB, Kovess-Masfety VK (2009). Psychological distress in first year university students: socioeconomic and academic stressors, mastery and social support in young men and women. Soc Psychiatry Psychiatr Epidemiol.

[20] Nerdrum P, Rustøen T, Rønnestad MH (2006). Student psychological distress: a psychometric study of 1750 Norwegian 1st-year undergraduate students. Scand J Educ Res.

[21] Coughlan M, Cronin P, Ryan F (2007). Step-by-step guide to critiquing research. Part 1: quantitative research. Br J Nurs.

[22] Johnson C, Christensen P (2004). Educational research: quantitative, qualitative and mixes approaches.

[23] Stallman HM (2010). Psychological distress in university students: a comparison with general population data. Aust Psychol.

[24] Verger P, Guagliardo V, Gilbert F, Rouillon F, Kovess-Masfety V (2010). Psychiatric disorders in students in six french universities: 12-month prevalence, comorbidity, impairment, and help-seeking. Soc Psychiatry Psychiatr Epidemiol.

[25] Bayram N, Bilgel N (2008). The prevalence and sociodemographic correlations of depression, anxiety and stress among a group of university students. Soc Psychiatry Psychiatr Epidemiol.

[26] Bernhardsdottir J, Vilhjalmsson R (2013). Psychological distress among university female students and their need for mental health services. J Psychiatr Ment Health Nurs.

[27] Pagel MD, Erdly WW, Becker J (1987). Social networks: we get by with (and in spite of) a little help from our friends. J Pers Soc Psychol.

[28] Shamsuddin K, Fadzil F, Ismail WSW (2013). Correlates of depression, anxiety and stress among Malaysian university students. Asian J Psychiatr.

[29] Buckley PF (2007). Dual diagnosis of substance abuse and severe mental illness: the scope of the problem. J Dual Diagn.

[30] Smith LL, Yan F, Charles M (2017). Exploring the link between substance use and mental health status: what can we learn from the self-medication theory?. J Health Care Poor Underserved.

[31] Damena T, Mossie A, Tesfaye M (2011). Khat chewing and mental distress: a community-based study, in Jimma city, South-western Ethiopia. Ethiop J Health Sci.

[32] Leahy CM, Peterson RF, Wilson IG, Newbury JW, Tonkin AL, Turnbull D (2010). Distress levels and self-reported treatment rates for medicine, law, psychology and mechanical engineering tertiary students: cross-sectional study. Aust N Z J Psychiatry.

[33] Volkow ND (2009). Substance use disorders in schizophrenia--clinical implications of comorbidity. Schizophr Bull.

[34] Porter K, Johnson PH, Petrillo J (2009). Priority health behaviors among South African undergraduate students. Int Electron J Health Educ.

[35] Aboalshamat K, Hou XY, Strodl E (2015). Psychological wellbeing status among medical and dental students in Makkah, Saudi Arabia: a cross-sectional study. Med Teach.

[36] Rezaei M, Khormali M, Akbarpour M, Sadeghniiat-Hagighi M, Shamsipour M (2018). Sleep quality and its association with psychological distress and sleep hygiene: a cross-sectional study among pre-clinical medical students. Sleep Sci.

[37] Tadom A, Cropley M (2008). Dysfunctional beliefs, stress and sleep disturbance in Fibromyalgia. Sleep Med.

